# Chronic Renal Failure Secondary to Unrecognized Neurogenic Bladder in A Child with Myelodysplasia

**Published:** 2017

**Authors:** Shameem AHMED, Siba Prosad PAUL

**Affiliations:** 1 Consultant Neurosurgeon, Department of Neurosurgery, Apollo Hospitals, (Unit: International Hospital), Guwahati, India.; 2 Consultant Pediatrician, Department of Pediatrics, Torbay Hospital, Torquay, UK.

**Keywords:** Neurogenic bladder, Lipomyelomeningocele, Chronic renal failure, Urinary tract infections, Clean intermittent catheterization, Myelodysplasia

## Abstract

Myelodysplasia includes a group of developmental anomalies resulting from defects that occur during neural tube closure. Urological morbidity in patients with myelodysplasia is significant and if not treated appropriately in a timely manner can potentially lead to progressive renal failure, requiring dialysis or transplantation. We report the case of a 13-year old girl with neurogenic bladder who presented chronic renal failure secondary to lipomyelomeningocele with retethering of cord. She was managed with urinary indwelling catheterization until optimization of renal function and then underwent detethering of cord with excision and repair of residual lipomeningomyelocele. Her renal parameters improved gradually over weeks and then were managed on self clean intermittent catheterization. The case emphasizes the need for considering retethering of spinal cord in children with myelodysplasia where symptoms of neurogenic bladder and recurrent urinary tract infections occur.

## Introduction

The term myelodysplasia describes a group of developmental anomalies resulting from defects that occur during neural tube closure (1). Neurological lesions may include spina bifida occulta, meningocele, lipomyelomeningocele, or myelomeningocele (1). Children born with spina bifida have lifelong complex health care needs. This necessitates involvement of specialists, general practitioners, and a multi-disciplinary team to deliver the complex care package (2). However, this much needed support network is often lacking in context of developing countries. Evolving pathologies may therefore remain undetected and health issues do get addressed symptomatically on an as required basis. 

Urological morbidity in patients with myelodysplasia is significant and the most common causes are pyelonephritis, renal dysfunction, and upper urinary tract deterioration. Myelodysplasia can cause significant urologic problems if not managed appropriately, which can potentially lead to progressive renal failure, requiring dialysis or transplantation (1). This case study describes a 13 years old girl with neurogenic bladder who presented with stigmata of chronic renal failure secondary to lipomyelomeningocele with retethering of spinal cord.

## Case Study

A 13-year girl was brought to the outpatient department in a teaching hospital in north-eastern India from a remote area with progressive painless weakness of both lower limbs over the previous 2 years. This was associated with history of urinary incontinence and recurrent urinary tract infections which was managed by a local general practitioner. Parents felt it was necessary to seek specialist advice as her condition was deteriorating and there was an increasing demand to support her mobility and medical needs. They gave history that the girl was born with a defect in lower back (lipomeningomyelocele) which was operated at 3 months of age as a curative intervention. She also had operative correction for bilateral Congenital Talipes Equinovarus. Parents also reported that the girl was treated for spontaneous fracture of bilateral tibia secondary to a minor injury. 

At presentation her vital parameters were stable. General physical examination revealed the girl to be pale with a puffy face. Urinary bladder was palpable with evidence of residual urine. Neurological examination revealed lower limb weakness and absent ankle jerk. She had also mild scoliosis to right side at lower lumbar region with tufts of hair. Blood investigation showed anaemia (haemoglobin 8.9g/dl), uraemia (urea 55.2 mg/ dl and creatinine 1.51 mg/dl) and evidence of urinary tract infection [UTI] (urine microscopy 35 x 10^6^/L of leucocytes and culture confirmed with *Escherichia coli*). Serial blood investigations highlighting renal parameters are highlighted in Table 1. Renal ultrasound scan showed dilated bladder with uneven wall changes in the form of trabeculation of the bladder wall as a sign of muscular hypertrophy. 

The girl was admitted to the paediatric ward and was initially managed conservatively with intravenous fluid, strict input and output monitoring, and intravenous Cefotaxime which was also found to be sensitive for the bacterial growth. A continuous indwelling Foley catheter was also inserted which was removed a few days later. Urology opinion was sought and urodynamic studies were planned for a later date. The girl was trained to do clean intermittent self catheterization (CIC) and it was advised that she does it at home every 3 hourly during the waking hours. 

**Fig 1.**
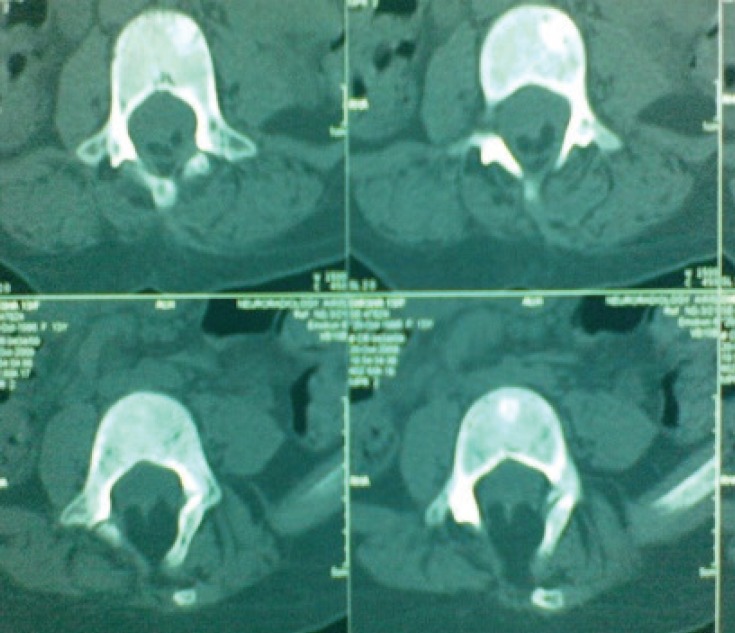
CT lumbar spine (Deficient posterior elements of L3-4

**Fig 2:**
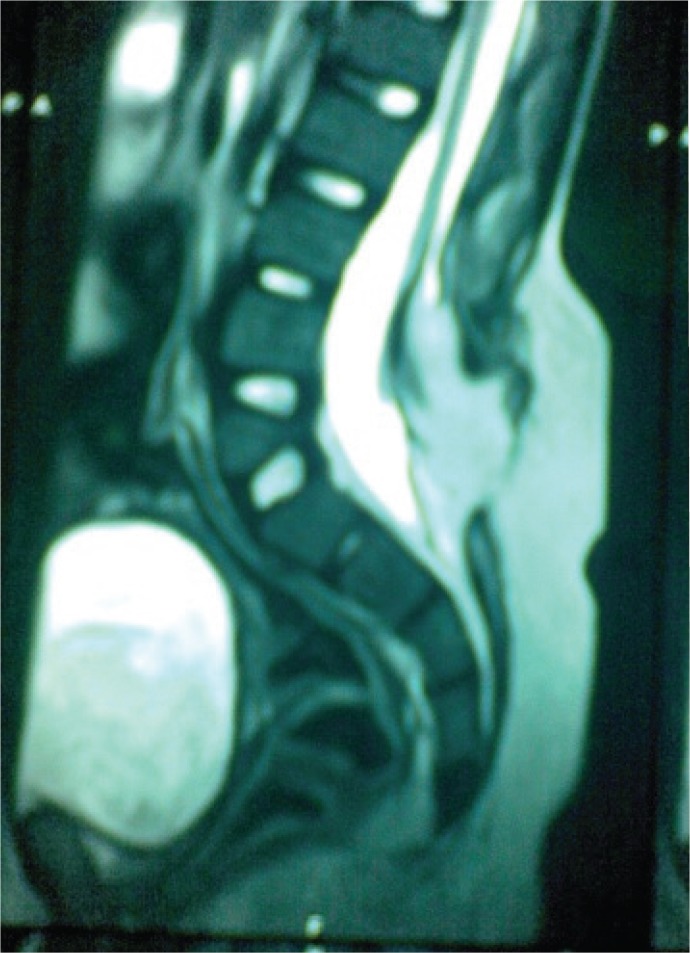
MRI Lumbosacral spine (Extradural fat component at 3rd to 5th lumbar vertebra with low lying conus at 4th lumbar vertebra

As no previous neuroimaging records were available, a CT scan of the spine was arranged the day after admission which showed spina bifida at 3rd and 4th lumbar vertebra (Fig 1). Neurosurgical advice was sought and MRI scan of the spine (Fig 2) revealed extradural fat component at 3rd to 5th lumbar vertebra with deficient posterior element at 3rd lumbar vertebra with low lying conus at 4th lumbar vertebra with evidence of retethering of cord. The deterioration in her clinical condition was considered to have occurred due to retethering of cord. On day 4 of inpatient admission she underwent detethering of cord with excision and repair of residual lipomeningomyelocele. In the postoperative period conservative treatment continued and renal parameters gradually improved with further uneventful hospital stay. The girl remains under the follow-up of paediatricians, urologists and neurosurgeons and is reported to be doing well with stable renal functions and no further UTI. 

**Table 1:** Serial Blood Results Showing Renal Deranged Parameters During the Admission

	**Sodium** **(135-145mmol/L)**	**Potassium** **(3.5-5.0mmol/L)**	**Urea** **(15-38mg/dl)**	**Creatinine** **(0.6 – 1.04mg/dl)**
**Day 1 **	147	5.6	55.2	1.51
**Day 2 **	145	5.2	46.8	1.46
**Day 4 **	143	4.9	42.6	1.39
**Day 5 **	141	5.4	40.8	1.29
**Day 7 **	140	5.0	36.6	1.24
**Day 10**	138	4.8	34.8	1.07
**Follow-up **	139	5.0	31.8	1.03

## Discussion

This case study described the challenges that many children and young people face with chronic progressive conditions with limited or no support network available in developing countries. Parents are left to decide when specialist health advice is necessary often financial constraints becoming the deciding factor. The girl in the case study had manifestations of a neurogenic bladder with sequelae of chronic renal failure secondary to lipomyelomeningocele. Although, neurogenic bladder is a common complication in patients with myelomeningocele but developing lipomeningocele is almost uncommon following its course. Early intervention would have improved her renal status and prevented progression to chronic renal failure. 

A Brazilian cohort study assessing the impact of an interdisciplinary approach in 192 children and adolescents with lower urinary tract dysfunction, 116/192 (60.4%) belonged to a neurogenic bladder dysfunction group (3). Myelomeningocele was the main diagnosis at baseline (n=90) followed by sacral agenesis (n=5), spine cord tumors (n=4), and others (n=17) (3). The study demonstrated that children with lower urinary tract dysfunction need an individualized approach, requires constant monitoring of clinical, laboratory and radiological imaging to minimize the risk of kidney damage (3). 

A postal survey of 169 clinics listed by the Spina Bifida Association of America, highlighted that there was a lack of consensus for the evaluation and management of bacteriuria in patients with spina bifida and neurogenic bladder (4). In children with spinal dysraphism, urodynamic studies should be done early to establish potential risk with an aim to maintaining low bladder pressures, decreasing risk of infection, and maintaining (2,5). 

CIC although necessary for most children with myelodysplasia can increase the incidence of febrile UTI (2,6). In a retrospective study of 76 myelodysplastic children over a 3-year follow-up period, 19 (25%) had one or more episodes of febrile UTI. It was demonstrated that low bladder compliance (<10 mL/cm H_2_O), detrusor overactivity, bladder trabeculation and the presence of vesicoureteral reflux to be significant factors in the incidence of febrile UTI (7). In a long term (11-years) follow-up study from Thailand, 36 children with myelomeningocele were treated in the first year of life (Group 1) were compared to 31 cases who were treated after the age of 3-years (Group 2), early treatment of neurogenic bladder using CIC yielded better results (7). In another study of 38 children from Japan evaluating the efficacy of CIC for urinary incontinence in myelodysplastic who children, socially acceptable continence was obtained in only 20 patients (52%) (8). 

Renal damage or failure can occur as a consequence of repeated UTIs leading to progressive renal scarring and damage or as a result of obstruction caused by inability to empty the bladder (2) Regular lifelong renal follow-up is required for patients with myelodysplasia as renal damage can occur slowly over a period of years or very rapidly. Renal failure although rare is known to occur despite maximal medical and surgical therapies, and dialysis or transplantation may be necessary in some patients (1,3). 

**In conclusion, **renal involvement in children with myelodysplasia is common and management of children with neurogenic bladder secondary to myelodysplasia needs the prevention of UTIs, minimise renal damage and achieve better urinary continence. Urodynamic studies are necessary for adequate management of children with myelodysplasia. Neurosurgical repair of lipomyelomeningocele at the time of diagnosis should be performed regardless of patient’s age or neurological function and is even more important in context of developing countries where there is risk of being lost to follow-up. It is important that health professsionals consider retethering of spinal cord as a possibility in children with melodysplasia where symptoms of neurogenic bladder and recurrent UTIs occur.

## References

[1] Favazza TF (2014). Myelodysplasia and Neurogenic Bladder Dysfunction.

[2] Larijani FJ, Moghtaderi M, Hajizadeh N, Assadi F (2013). Preventing Kidney Injury in Children with Neurogenic Bladder Dysfunction. Int J Prev Med.

[3] de Azevedo RV, Oliveira EA, Vasconcelos MM (2014). Impact of an interdisciplinary approach in children and adolescents with lower urinary tract dysfunction (LUTD). J Bras Nefrol.

[4] Elliott SP, Villar R, Duncan B (2005). Bacteriuria management and urological evaluation of patients with spina bifida and neurogenic bladder: a multicenter survey. J Urol.

[5] Danforth TL, Ginsberg DA (2014). Neurogenic lower urinary tract dysfunction: how, when, and with which patients do we use urodynamics?. Urol Clin North Am.

[6] Seki N, Masuda K, Kinukawa N, Senoh K, Naito S (2004). Risk factors for febrile urinary tract infection in children with myelodysplasia treated by clean intermittent catheterization. Int J Urol.

[7] Kochakarn W, Ratana-Olarn K, Lertsithichai P, Roongreungsilp U (2004). Follow-up of long-term treatment with clean intermittent catheterization for neurogenic bladder in children. Asian J Surg.

[8] Obara K, Mizusawa T, Isahaya E (2010). Efficacy of Clean Intermittent Catheterization for Urinary Incontinence in Children with Neurogenic Bladder Dysfunction Secondary to Myelodysplasia. Low Urin Tract Symptoms.

